# The Relationship between the Browning Index, Total Phenolics, Color, and Antioxidant Activity of Polish-Originated Honey Samples

**DOI:** 10.3390/foods10050967

**Published:** 2021-04-28

**Authors:** Małgorzata Starowicz, Anita Ostaszyk, Henryk Zieliński

**Affiliations:** 1 Department of Chemistry and Biodynamics of Food, Institute of Animal Reproduction and Food Research, Polish Academy of Sciences, 10-784 Olsztyn, Poland; h.zielinski@pan.olsztyn.pl; 2 Sensory Laboratory, Institute of Animal Reproduction and Food Research, Polish Academy of Sciences, 10-784 Olsztyn, Poland; a.ostaszyk@pan.olsztyn.pl

**Keywords:** bee product, browning index, buckwheat honey, L*a*b* parameters, scavenging radicals, total phenolics

## Abstract

Honey is a source of sugars, amino acids/proteins, and polyphenols, which are the main substrates and reactants in the Maillard reaction. Several bioactive molecules are formed and sequestered to the brown polymeric melanoidins, resulting in a gain and loss of antioxidant function in honey. Therefore, the relationships between the browning index and total phenolic contents, color, and antioxidant activity of Polish-originated honeys, namely acacia, buckwheat, heather, linden, multiflorous, and rapeseed, obtained from three local beekeepers, were addressed in this study. The Total Phenolic Content data showed the following order: buckwheat > heather > acacia > multiflorous > linden > rapeseed. The buckwheat honey also had the highest ability to scavenge free radicals in the range of 207.1–289.3 and 40.9–52.3 µmol Trolox g^−1^, provided by Antioxidant Compounds Water-soluble and Ferric Reducing Antioxidant Power assays, respectively. Furthermore, a higher degree of browning was observed in dark-colored honey, such as buckwheat (3.1) and heather (1.35 mAU), than in light ones. Moreover, L* and b* parameters had a greater value in the honey of multiflorous, linden, and rapeseed, and a* was higher in buckwheat and heather. The variables of browning and TPC, ACW, and FRAP were positively correlated with each other. It can be concluded that the browning index strongly contributed to parameters of honey appearance, bioactive compound content, and antioxidant activity.

## 1. Introduction

Honey is a sweet and aromatic natural food product that is widely consumed. According to the consumer study of Kopała, Kuźnicka, and Balcerak [1], in Poland, the most preferable honeys are the light ones, including multiflorous, linden, acacia, rapeseed. From the dark ones, buckwheat honey has the highest consumer acceptance. Honey is mainly a composition of sugars, but is also a good source of varied molecules, flavonoids, and phenolic acids, with high biological and antioxidant activity [2]. Honey is also presented as a “reaction pot”, where the main honey constituents—sugars, amino acids/proteins, and polyphenols—are substrates and reactants in the Maillard reaction. During the process, several bioactive molecules, modified proteins, and protein-polyphenol complexes are formed and sequestered to the brown polymeric melanoidins, resulting in a gain and loss of antioxidant capacity and antibacterial function in honey [3]. Melanoidins are compounds generated in the late stages of the Maillard reaction during food processing and preservation [4]. Brudzynski et al. [5] explained that this could be related to the reaction of polyphenols with proteins, and it could be connected with polyphenol-type complexes and melanoidin formation. Nowadays, melanoidins have attracted a lot of attention, not only as a functional food ingredient, but also as a potentially healthy dietary supplement [6]. The Maillard reaction and formation of melanoidins in honey is a key mechanism underlying honey’s antibacterial and antioxidant activities [7,8]. In our study, the melanoidin content will be included in the browning index.

The antioxidant activity has already been measured using different methods, such as 1,1 diphenyl-2-picrylhydrazyl (DPPH), ABTS (2,2’-azino-bis(3-ethylbenzothiazoline-6-sulfonic acid), ferric reducing antioxidant power (FRAP), photochemiluminescence assay for antioxidant compounds, water-soluble (ACW), and lipid-soluble (ACL) modes [9,10,11]. Recently, Dżugan, Tomczyk, Sowa, and Grabek-Lejko [12] suggested that antioxidant activity can be a useful parameter for determining the botanical origin of honey. Furthermore, Socha et al. [13] found a linear correlation between phenolic content and antioxidant activity in different varieties of Polish honeys. Moreover, the high antioxidant activity of honey might be correlated with its health effects against e.g., cancer, infectious diseases, and wounds [14,15]. However, the mechanism of the anti-cancer activity of honey as a chemopreventive and therapeutic agent has not been completely understood. The possible mechanisms are due to its apoptotic, antiproliferative, antitumor necrosis factor (anti-TNF), antioxidant, anti-inflammatory, estrogenic, and immunomodulatory activities [16]. In other studies, Schramm et al. [17] proved that intake of buckwheat honey increases phenolic content in plasma and, therefore, its antioxidant status. It is already known that honey composition, color, and flavor depend mostly on the type of flowers used by bees, the geographical origin, and climate changes. The influence of these aspects on honey quality is briefly described in the review of da Silva et al. [18]. Significant differences in honey quality were also recognized between regions of the same country [19]. Moreover, da Silva et al. [18] pointed out that factors involved with the production process, like processing, packaging, and storage period, could affect the quality of honey. This could mean that not only honey type, but also the production technique is an important aspect to achieve high-quality honey.

Therefore, the objective of this study was to describe the relationships between browning index and total phenolic contents, color, and antioxidant activity of Polish-originated honeys, namely acacia, buckwheat, heather, linden, multiflorous, and rapeseed. The formation of brown pigments e.g., melanoidins, was measured arbitrarily at 450 nm. Then, the Folin–Ciocalteau assay was used for the determination of the total phenolic content (TPC). The color of the honey was obtained considering three parameters, L*, a*, and b*, of the CIELAB (Commission Internationale de l’Eclairage) system. The color was also characterized by professional panelists during a sensory session. The antioxidant activity was measured by photochemiluminescence assay in the mode of water-soluble antioxidant compounds (ACW) and by ferric reducing antioxidant power (FRAP) assay. Moreover, differences/similarities between the honey types and three honey producers were also studied using principal component analysis (PCA), and were widely discussed.

## 2. Materials and Methods

### 2.1. Chemicals

6-Hydroxy-2,5,7,8-tetramethylchroman2-carboxylic acid (Trolox); 2,4,6-tris(2-pyridyl)-1,3,5-triazine (TPTZ); iron (III) chloride hexahydrate (FeCl_3_·6H_2_O); sodium acetate; and acetic and hydrochloric acids were purchased from Sigma (Sigma Chemical Co., St. Louis, MO, USA). A PCL kit for hydrophilic antioxidants (ACW) was purchased from Analytik Jena (Jena, Germany). Ethanol was purchased from POCH S.A. (Gliwice, Poland). Water was purified by a Milli-Q system (Millipore, Burlington, MA, USA).

### 2.2. The Origin of Honey Samples

Honey samples were collected during the 2017 season in the Warmia and Mazury region (northeast part of Poland) and supplied from three local and professional beekeepers, who are representatives of Culinary Heritage of Warmia, Mazury, and Powiśle. Honey variety was declared by the producers according to the location of the beehive and available floral source: acacia (*Acacia* Mill.), buckwheat (*Fagopyrum esculentum* Moench.), heather (*Calluna vulgaris* Hull), linden (*Tilia cordata* Mill.), rapeseed (*Brassica napus* L.), and multiflorous. Honey jars were kept in a dark place at room temperature (20–22 °C) before analysis.

### 2.3. Determination of Browning Index

The browning index was estimated as the absorbance at 450 nm, according to the methodology described by Brudzynski and Miotto [3]. The assay was performed in a microplate reader (Tecan M1000 Infinite PRO, Basel, Switzerland). The measurements for each honey were repeated three times. The results of semi-quantitative measurements were expressed as arbitrary absorbance units (AU).

### 2.4. Determination of Color by CIE Lab System and Sensory Panel

The honey color was measured considering three parameters, L*, a*, and b*, of the CIELAB system using the equipment of ColorFlex (Hunterlab, Reston, VA, USA). While L* is an index giving information about lightness, a* is positive in reddish colors and negative in greenish colors, and b* is positive in yellowish colors and negative in bluish colors [20]. Before the analysis of color, the honeys were liquefied in a water bath at a temperature up to 40 °C to achieve the transparent samples without any dilution. Values were the mean of at least three replicates.

The evaluation of color was also carried out by a seven-person team selected, trained, and monitored following ISO 8586:2012 [21], with appropriate methodological preparation and experience in sensory profiling of various products. The assessments were carried out in the Sensory Laboratory of the Institute of Animal Reproduction and Food Research of the Polish Academy of Sciences in Olsztyn, meeting the requirements of ISO 8589:2007 [22]. Then, the color was assessed on a linear scale (corresponding to 10 conventional units–c.u.). The boundary terms of the scale for the color were: 0—light to 10—dark in the scale of yellow (in the case of acacia, linden, rapeseed, and multiflorous honey) and brown (heather and buckwheat honey).

### 2.5. Determination of Honey’s Antioxidant Activity by ACW and FRAP Assays

Honey samples were extracted according to the methodology described by Wilczyńska [9] with 96% ethanol. Then, extracts were stored at −80 °C until the analysis. To determine antioxidant capacity, honey samples were analyzed using a photochemiluminescence assay in the mode of antioxidant capacity in water-soluble substances (ACW) and ferric reducing antioxidant potential (FRAP) assay.

The ACW method is used to measure the ability of antioxidants from extracts to scavenge superoxide anion radicals (O_2_^−^). Therefore, the ACW measurement was performed using PHOTOCHEM apparatus (Analytik Jena, Jena, Germany) according to protocols elaborated by Zieliński, Zielińska, and Kostyra [23]. The lag time (250 s) for the ACW test was used as a free radical scavenging activity. The antioxidant activity was calculated by comparing it with the Trolox standard curve (0.5–3 nmol). The antioxidant capacity was calculated as μmol Trolox equivalents per gram of honey (μmol Trolox g^−1^).

The FRAP method is based on the reduction of ferric ions by antioxidant compounds. The FRAP assay was conducted according to the experiment of Horszwald and Andlauer [24]. The sample absorbance was measured at 593 nm after 5 min of reaction with a microplate reader (Tecan M1000 Infinite PRO, Männedorf, Switzerland). The antioxidant capacity was calculated as μmol Trolox equivalents per gram of honey (mmol Trolox g^−1^). The antioxidant tests were performed in triplicate for each sample.

### 2.6. Determination of the Total Phenolic Content (TPC)

The measurements of TPCs were performed in microplates (Infinite M1000 Pro Multimode Microplate Reader, Tecan, Männedorf, Switzerland) using the procedure of Horszwald and Andlauer [24]. The TPC assay was performed in microplates, and aliquots of 15 μL of methanol extracts were placed in microplate wells. Subsequently, 250 μL of the Folin–Ciocalteu reagent (previously diluted with water, 1:15, *v/v*) were added, and the mixture was incubated for 10 min in the dark at room temperature. Then, 25 μL of 20% sodium carbonate were added to each well. The absorbance of the mixture in the measurement of TPC was evaluated with the Folin–Ciocalteu reagent at 755 nm after 5 min of reaction in microplates. The results were calculated according to prepared standard curves of gallic acid in the range of 0.03–1.0 mg L^−1^ and presented as milligrams of gallic acid equivalent (GAE) per gram of sample (mg GAE g^−1^). Three replicates were analyzed for each honey type.

### 2.7. Statistical Analysis

The data are presented as mean values and standard deviations of triplicate measurements. The differences between the samples were analyzed by a one-way ANOVA with Tukey’s multiple comparison test (*p* < 0.05) using STATISTICA 13.1 (StatSoft Inc., Tulsa, OK, USA). The principal component analysis (PCA) was performed using Addinsoft (XLSTAT ver. 19.01, Paris, France).

## 3. Results and Discussion

### 3.1. The Browning Index of Honey Samples

**Figure 1:**
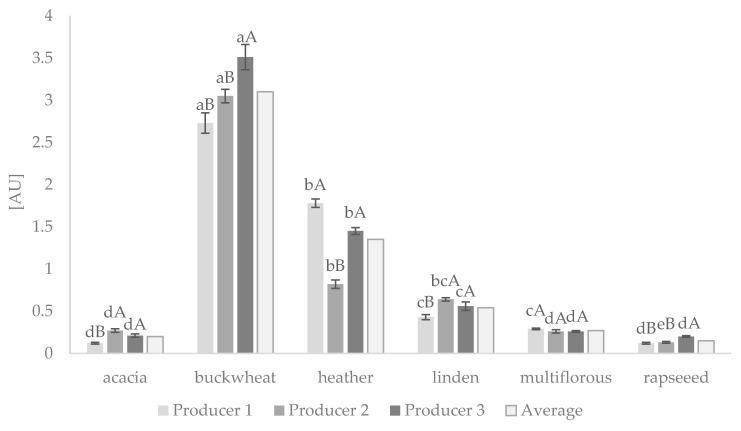
The degree of browning of honey samples from three different producers. The peaks are presented as the mean ± standard deviation. ^a–e^ Values followed by different letters are significantly different between each type of honey (*p* < 0.05); ^A,B^ values followed by different letters are significantly different between producers 1, 2, and 3 (*p* < 0.05), as determined by the Tukey’s multiple comparisons test.

The degree of browning of honey from three producers is shown in Figure 1. The highest degree of browning was observed in dark-colored honeys, such as buckwheat (3.73–3.51 AU), followed by heather (0.82–1.18 AU). The average browning in light honeys, like rapeseed, acacia, multiflorous, and linden, was 20-fold, 15-fold, 11-fold, and almost six-fold lower, respectively, compared to buckwheat honey. It was noted that, amongst the three producers of Polish-originated honeys, the degree of browning varied significantly at least from one producer (*p* < 0.05). The only browning index in multiflorous honey was not dependent on the producer localization. The average degree of browning that could be related to melanoidin content in Polish-originated acacia honey from the three different producers was the same as in India-originated acacia ones, while multiflorous honey was twice as rich [25]. Beretta et al. [26] presented lower color intensity (Abs_450_) for buckwheat honey, but this could be related to the measuring of 50% diluted honey samples.

Our results confirmed that the Maillard reaction and melanoidin formation occurs in unheated Polish-originated honeys. It was reported that concentrations of catalytic sugars and a pool of free amino acids promote and facilitate the final stage of the reaction, resulting in the formation of brown melanoidins of a high molecular weight that exhibit antioxidant activity [4,27]. Moreover, it was proven that heat treatment of honey increased melanoidin formation, and its appearance coincided with the enhanced antioxidant activity [3]. Brudzynski and Miotto [3] suggested that phenolic compounds are involved in melanoidin formation and provide melanoidins with antioxidant activity. Therefore, in our study, it was of increased concern to find out the relationship between the browning index content and markers of honey quality, such as color, TPCs, and antioxidant capacity, measured by two independent ACW and FRAP assays.

### 3.2. Honey Color Determined by CIELAB System and Sensory Panel

Color is the first attractive attribute of honey, and, as such, it is very important for commercialization. It is an important parameter in the quality, acceptance, and preference of consumers. Color can be measured using the CIELAB system. It expresses color as three values: L* for lightness and a* and b* for the red, green, blue, and yellow colors. The lightness of honey plays an important role due to consumer preferences. According to our consumer survey (data not published), respondents preferred light honeys (58.2%) over dark ones (14.9%). However, color is mainly determined by its botanical origin. The Codex Alimentarius Committee on Sugars [28] stipulates that the color of honey should be nearly colorless to dark brown. The same observations were described by Šedík, Prokeinová, and Horská [29], in the study of whom a group of Slovakian youths (age 18–30 years old) claimed to prefer significantly light honeys, such as acacia and linden types.

The studied Polish honeys according to color measurements can be divided into light, medium, or dark, differing in the number of melanoidins produced. Light-colored honeys, like acacia and rapeseed, produced the lowest amounts of melanoidins, medium amounts were noted in multiflorous and linden, and the highest content was shown in buckwheat and heather (Figure 1). Moreover, based on the color measurements using the CIELAB method, a similar picture was obtained (Table 1).

The highest lightness values were ascribed to rapeseed and multiflorous honeys originated from the three producers, whereas the lowest ones were noted for buckwheat and heather honeys. In our study, the lightness values for acacia honeys from producer 1 and 2 were significantly lower compared to that of producer 3 and the available literature, whereas comparable results were observed for multiflorous honey [25]. Moreover, our multiflorous and rapeseed honey was significantly lighter (higher L* values) than e.g., citrus honey from Egypt [30]. It was found out that the high intensity of honey color might be related to a high concentration of Maillard-derived polymers—melanoidins [3]. The brownish color and medical odor were one of the main descriptors, which allowed the distinguishing of significant buckwheat honeys (*p* < 0.05).

**Figure 2:**
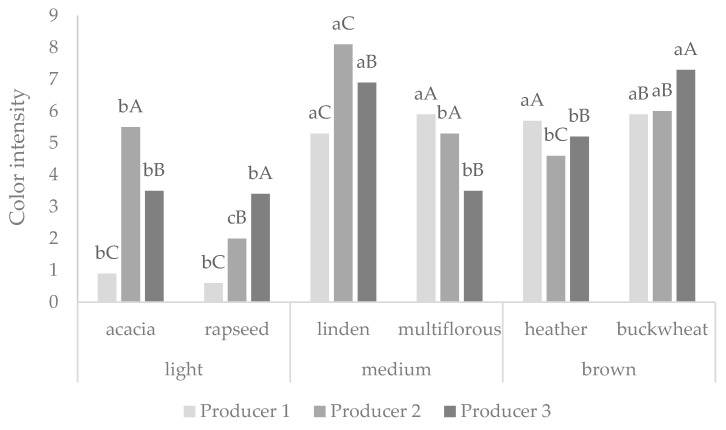
Color intensity measured by the sensorial panel. Values are presented as the mean ± standard deviation. ^a,b^ Values followed by different letters in the same column are significantly different (*p* < 0.05), as determined by the Tukey’s multiple comparisons test. ^A–C^ Values followed by different letters in the same column are significantly different (*p* < 0.05), as determined by the Tukey’s multiple comparisons test.

Pita-Calvo, Guerra-Rodríguez, and Vázquez [31] classified color measurement as the main analysis to characterize honey quality. The evaluation of color intensity provided by a seven-person team is shown in Figure 2. The results provided by panelists confirmed the data on the honey’s color by using the CIELAB method. The rapeseed honey could be classified as light honey and buckwheat as dark, and this notification was in agreement with that provided by Dżugan et al. [12].

### 3.3. Total Phenolic Content (TPC) in Different Honey Types

The total phenolic content determined by the Folin–Ciocalteu method varied greatly among the honey types, as shown in Table 2.

Heather and buckwheat honeys were characterized by a significantly higher content of phenolic compounds (on average, 159.2 and 141.1 mg GAE 100 g^−1^, respectively) compared to the other tested varieties (*p* < 0.05). The TPC of multiflorous, rapeseed, and acacia honeys was determined to be in the range of 37.7–45.0, 13.5–28.9, 42.3–65.4 mg GAE 100 g^−1^, respectively. It was noted that, amongst the three producers of Polish-originated honeys, the TPC varied significantly at least from one producer (*p* < 0.05). The TPC values for multiflorous honey were similar to those obtained in a previous study by Sawicki, Bączek, and Starowicz [32], about 49 mg GAE 100 g^−1^, but significantly higher for acacia honey than reported by Attanzio et al. [33] for Sicilian honey. The correlation coefficients between the TPC and browning index of honeys obtained from producers 1, 2, and 3 were r = 0.96, 0.36, and 0.89, respectively. Therefore, an average TPC was generally positively correlated with an average degree of browning (r = 0.80), while a weak correlation was noted between the average TPC and color provided by the sensory panel (r = 0.40). Except from producer 2, the rank of the TPC in honey samples was as follows: buckwheat > heather > acacia > multiflorous > linden > rapeseed.

Our findings are in agreement with data collected by Tomczyk, Tarapatskyy, and Dżugan [11], who established the TPC in Polish honey samples on the same level. The high TPC in buckwheat and heather honey was also noted by Wesołowska and Dżugan [34]. The obtained results were compared with other authors’ findings for selected honey types. Wilczyńska [10] found the total phenolic content for Polish honeys in the range of 17.8 (rapeseed) to 189.5 (heather) mg GAE 100 g^−1^. A twice higher TPC in multiflorous honey from Burkina Faso was noted by Meda et al. [35] compared to the results provided by our study.

The results provided in our study indicate a link between the TPC and browning of honeys. Brudzynski and Miotto [3] indicated that phenolic compounds are involved in honey browning formation in a way that includes their attachment to the existing high molecular weight polymer. This process occurs in unheated honeys, but is greatly facilitated by high temperatures, and it has an impact on the antioxidant capacity of honeys. In other words, the antioxidant capacity of honey may be formed by not only phenolic compounds, but also by the brown pigment formation with the contribution of phenolics.

### 3.4. Antioxidant Capacity of Honey Samples Provided by ACW and FRAP Assays

In the present study, the photochemiluminescence technique (ACW) and FRAP method for reducing antioxidant power were applied for the determination of the antioxidant capacity of the honey samples. The ACW assay is based on the photo-induced autoxidation inhibition of luminol by antioxidants, mediated from the radical anion superoxide, and is suitable to measure the radical scavenging properties of single antioxidants, as well as more complex food systems [36]. The FRAP assay provides the antioxidant potential of the samples by measuring the reduction of ferric iron (Fe^3+^) to ferrous iron (Fe^2+^).

The superoxide anion radical scavenging capacity of honey varied from 26.94 to 255.40 (mean values) in the ACW system (Table 3). The highest antioxidant capacity was noted for buckwheat honeys in the range from 207.1–289.8 µmol Trolox g^−1^. Jasicka-Misiak et al. [37] indicated that abscisic acid could be a potential biomarker for determining the origin of heather and buckwheat, and could significantly influence the biological potential of these two types of honey. The lowest ACW values were observed in acacia (33.5–37.6 µmol Trolox g^−1^) and multiflorous (26.2–27.7 µmol Trolox g^−1^) honey. These findings were in agreement with earlier research conducted by Wilczyńska [10] and Wesołowska and Dżugan [34], where a similar tendency was observed. The average FRAP values ranged from 5.29 mmol Trolox g^−1^ (rapeseed honey) to 47.13 mmol Trolox g^−1^ (buckwheat honey). These results are in agreement with Socha et al. [13], who also determined the lowest antioxidant activity for rapeseed honey and the highest values for buckwheat honey. Wesołowska and Dżugan [34] obtained slightly higher FRAP results for multiflorous and rapeseed honeys. However, it should be pointed out that the antioxidant capacity of honeys depends on several factors, such as geographic origin, collection season, mode of storage, bee species, and even interactions between chemical compounds and enzymes in the honey.

The results of ACW obtained for honeys from producers 1, 2, and 3 were highly correlated with the respective FRAP data (r = 0.91, r = 0.87, and r = 0.72). However, what is more important is that the antioxidant capacity of honeys obtained from producers 1, 2, and 3 was highly positively correlated with the TPC (r = 0.80, r = 0.58, and r = 0.78, respectively) and melanoidin content (r = 0.92, r = 0.95, and r = 0.97, respectively). Similar highly positive correlations were also noted between the FRAP and TPC and melanoidin content, thus confirming the contribution of melanoidins to the formation of the antioxidant capacity formed by water-soluble antioxidants. Chua et al. [38] found that the content of water-soluble vitamins was well-correlated with antioxidant activity. Similarly, Beretta et al. [26] reported a high value of the correlation coefficient (r = 0.93), describing the relationship between phenol content and color intensity characterized as absorbance at 450 nm. Moreover, the correlation between phenolic content and antioxidant activity of the honey samples was frequently examined. Wilczyńska [10] also presented a strong positive correlation between the antioxidant activity and total phenolic content (r = 0.74 for the TPC vs. DPPH, r = 0.55 for TPC vs. ABTS). Meda et al. [35] found a correlation between radical scavenging activity and the total phenolic content, while other authors found it stronger [26,39,40]. On the other hand, Stagos et al. [41] did not find any statistical correlation between DPPH or ABTS tests and TPC. This means that phenolics are one of the main components responsible for the antioxidant activity of honeys. To describe the ability to scavenge free radicals, the FRAP assay is the most appropriate in comparison to other methods (e.g., DPPH, ABTS, and ORAC).

### 3.5. Results of Principal Component Analysis (PCA) for Honey Samples

**Figure 3:**
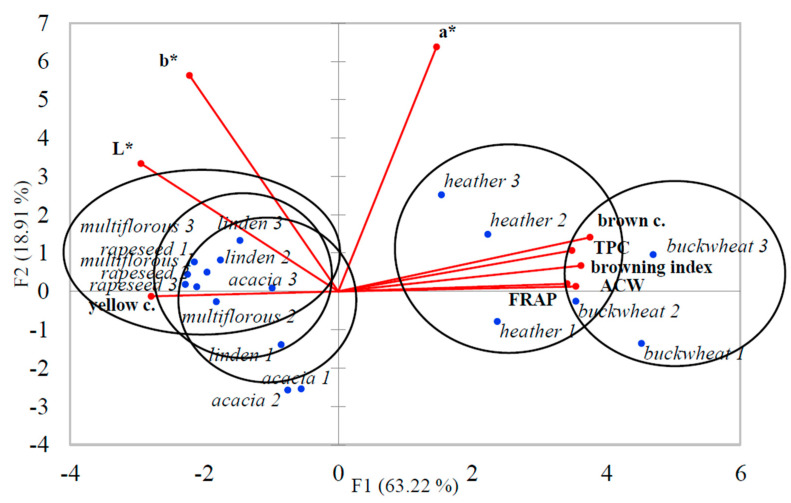
PCA biplot for honey samples from all producers (1–3) with nine variables (browning index, color parameters: L*, a*, b*, yellow and brown color, TPC, ACW, and FRAP).

The PCA was prepared to find similarities and differences between melanoidin content, antioxidant activity (ACW and FRAP assays), total phenolic content (TPC), and color obtained in the CIELAB system and during sensory evaluation (brown and yellow color) obtained by the panel. The combination of PCA accounted for 63.22% for F1 and 18.91% for F2 of the variance in the data. The first two principal components (F1 and F2) explained 82.13% of the total data variance. Karabagias and Karabournioti [30] achieved a slightly higher total data variance, which could be associated with a higher amount of analyses performed by these authors. The correlations between the original variables and the obtained principal components are shown in Figure 3. Each of the variables is represented by a vector. The direction and lengths of the vectors indicate to what extent the given variables affected the principal components. The browning index, ACW and FRAP, TPC, as well as the brown color were highly correlated with buckwheat and heather honeys, opposite of multiflorous, linden, acacia, and rapeseed honeys, which were correlated with yellow color and the lightness value, L*. The most correlated with value b* were light honeys and, with value a*, the dark honeys. As was previously reported by Kuś et al. [42], we also proved that CIELAB color parameters might be helpful as a support for honey identification or classification.

Using PCA, Kaygusuz et al. [43] successfully distinguished five types of honey samples: heather, oak, flower, pine, and chestnut. In our case, we were able to differentiate light honey types (acacia, linden, multiflorous) from the dark ones (heather and buckwheat). Therefore, it can be concluded that PCA is a useful chemometric tool to describe honey botanical origin.

## 4. Conclusions

Evidence is growing that honey may have antioxidant potential through scavenging the reactive oxygen species and reducing the prooxidative metal cations. We have demonstrated that the Maillard reaction and melanoidin formation occurred in unheated Polish-originated honey, and brown pigment formation was strongly correlated with antioxidant potential. Generally, dark honeys showed better antioxidant capacity and higher melanoidin content compared to light honeys. Moreover, our study indicates a link between the TPC and melanoidin formation in honeys. Among the Polish-originated honey, buckwheat, heather, and linden showed the highest ability to scavenge superoxide anion radicals. A highly positive correlation noted between the browning index and antioxidant capacity provided by the ACW assay confirmed the contribution of brown pigments to the formation of antioxidant capacity formed by water-soluble antioxidants. A better understanding of these findings will help reveal the mechanisms of honey functions and predict honey’s effect in different biological systems. As was demonstrated by Vela, de Lorenzo, and Pérez [44], honey, a source of antioxidants, could be used further to avoid the enzymatic browning of fruits and fruit products.

## Figures and Tables

**Table 1:** Color measurement in different types of honey using the CIELAB method.

Type of Honey	Producer 1			Producer 2			Producer 3		
	L*	a*	b*	L*	a*	b*	L*	a*	b*
Acacia	8.70 ± 0.03 ^e^	–2.12 ± 0.08 ^f^	6.87 ± 0.16 ^e^	9.17 ± 0.03 ^e^	–1.38 ± 0.16 ^f^	3.08 ± 0.13 ^f^	47.52 ± 0.05 ^c^	4.02 ± 0.03 ^e^	31.51 ± 0.09 ^d^
Buckwheat	3.38 ± 0.07 ^f^	1.89 ± 0.19 ^d^	3.86 ± 0.07 ^f^	8.40 ± 0.24 ^f^	8.68 ± 0.08 ^b^	9.21 ± 0.30 ^e^	12.29 ± 0.10 ^f^	12.78 ± 0.26 ^b^	17.17 ± 0.11 ^f^
Heather	9.24 ± 0.01 ^d^	3.87 ± 0.10 ^c^	12.45 ± 0.09 ^d^	20.38 ± 0.12 ^d^	14.82 ± 0.12 ^a^	26.70 ± 0.15 ^d^	33.97 ± 0.07 ^d^	16.86 ± 0.04 ^a^	40.20 ± 0.08 ^b^
Linden	14.58 ± 0.18 ^c^	1.61 ± 0.19 ^e^	19.54 ± 0.28 ^c^	48.71 ± 0.04 ^b^	6.53 ± 0.09 ^c^	41.43 ± 0.31 ^a^	31.77 ± 0.06 ^e^	9.47 ± 0.03 ^c^	50.22 ± 0.23 ^a^
Multiflorous	57.29 ± 0.11 ^b^	5.12 ± 0.13 ^b^	34.90 ± 0.20 ^a^	40.51 ± 0.06 ^c^	3.50 ± 0.06 ^e^	29.94 ± 0.17 ^c^	56.26 ± 0.12 ^b^	6.56 ± 0.02 ^d^	37.75 ± 0.03 ^c^
Rapeseed	59.45 ± 0.01 ^a^	6.07 ± 0.02 ^a^	33.33 ± 0.02 ^b^	59.45 ± 0.01 ^a^	3.63 ± 0.05 ^d^	32.21 ± 0.08 ^b^	72.17 ± 0.20 ^a^	3.36 ± 0.06 ^f^	29.34 ± 0.11 ^e^

Values are presented as the mean ± standard deviation. ^a–f^ Values followed by different letters in the same column are significantly different (*p* < 0.05), as determined by the Tukey’s multiple comparisons test.

**Table 2:** The total phenolic content (TPC) in honey samples.

Type of Honey	TPC			Average Content
	(mg GAE 100 g^−1^)			
	Producer 1	Producer 2	Producer 3	
Acacia	42.3 ± 7.40 ^cC^	58.9 ± 2.56 ^cB^	65.4 ± 1.39 ^cA^	55.5
Buckwheat	170.3 ± 1.91 ^aA^	97.2 ± 5.12 ^bC^	155.7 ± 2.09 ^aB^	141.1
Heather	156.7 ± 4.61 ^bB^	195.1 ± 10.25 ^aA^	125.8 ± 5.20 ^bC^	159.2
Linden	24.7 ± 1.63 ^dB^	32.8 ± 0.55 ^dA^	25.9 ± 0.15 ^eB^	27.8
Multiflorous	37.7 ± 3.64 ^cA^	31.4 ± 0.12 ^eB^	45.0 ± 0.5 ^dA^	38.0
Rapeseed	13.5 ± 0.78 ^eC^	17.5 ± 0.50 ^eB^	28.9 ± 1.36 ^eA^	19.9

Values are presented as the mean ± standard deviation. ^a–e^ Values followed by different letters in the same column are significantly different (*p* < 0.05); ^A–C^ values followed by different letters in the same row are significantly different (*p* < 0.05), as determined by the Tukey’s multiple comparisons test.

**Table 3:** Data on antioxidant activity measured with ACW and FRAP assays.

Type of Honey	ACW				FRAP			Average
	(µmol Trolox g^−1^)				(mmol Trolox g^−1^)			
	Producer 1	Producer 2	Producer 3	Average	Producer 1	Producer 2	Producer 3	
Acacia	33.5 ± 0.40 ^eB^	36.0 ± 2.61 ^eA^	37.6 ± 1.93 ^eA^	35.70	18.5 ± 0.36 ^dC^	20.0 ± 0.01 ^dB^	35.1 ± 1.66 ^bA^	24.53
Buckwheat	289.8 ± 14.1 ^aA^	207.1 ± 22.9 ^aC^	269.3 ± 5.50 ^aB^	255.40	52.3 ± 1.02 ^aA^	40.9 ± 0.15 ^aC^	48.2 ± 0.09 ^aB^	47.13
Heather	94.1 ± 5.18 ^bB^	119.9 ± 2.51 ^bA^	72.5 ± 0.11 ^bC^	95.50	29.7 ± 0.01 ^fB^	32.0 ± 0.05 ^bA^	33.0 ± 0.01 ^aB^	31.58
Linden	61.6 ± 0.22 ^cB^	67.7 ± 0.12 ^cA^	64.5 ± 2.41 ^cB^	64.60	27.2 ± 0.89 ^cB^	25.4 ± 0.80 ^cA^	27.9 ± 0.04 ^cB^	26.83
Multiflorous	26.2 ± 1.32 ^fA^	27.7 ± 0.95 ^fA^	26.9 ± 0.23 ^fA^	26.94	10.8 ± 0.05 ^eB^	15.9 ± 0.04 ^eA^	11.6 ± 0.01 ^dB^	12.77
Rapeseed	40.3 ± 1.82 ^dB^	43.5 ± 0.72 ^dA^	46.1 ± 2.05 ^dA^	43.30	4.85 ± 0.08 ^eC^	5.33 ± 0.01 ^fB^	5.69 ± 0.02 ^eA^	5.29

Values are presented as the mean ± standard deviation. ^a–f^ Values followed by different letters in the same column are significantly different (*p* < 0.05); ^A–C^ values followed by different letters in the same row are significantly different (*p* < 0.05), as determined by the Tukey’s multiple comparisons test. The average value from the three producers.
